# Demographic differences in facial anthropometric data from 3D scans and implications for respirator fit

**DOI:** 10.1093/annweh/wxaf012

**Published:** 2025-03-19

**Authors:** Kayna Hobbs-Murphy, William J Brazile, Kristen Morris, John Rosecrance

**Affiliations:** Department of Design and Merchandising, Colorado State University, 1574 Campus Delivery, Fort Collins, CO 80523-1574, United States; Department of Environmental and Radiological Health Sciences, Colorado State University, 1681 Campus Delivery, Fort Collins, CO 80523-1681, United States; Department of Design and Merchandising, Colorado State University, 1574 Campus Delivery, Fort Collins, CO 80523-1574, United States; Department of Environmental and Radiological Health Sciences, Colorado State University, 1681 Campus Delivery, Fort Collins, CO 80523-1681, United States

**Keywords:** demographic differences, facial anthropometry, respirator fit, 3-dimensional body scanning

## Abstract

**Objective:**

In response to limitations in foundational anthropometric research efforts as well as the increasingly diversifying workforce, researchers have attempted to define the presence or absence of differences in respirator-related facial measurements between different demographic groups. The purpose of the present study was to assess the presence of differences in facial measurements from 3D scans related to respirator fit, based on demographic factors of gender, race/ethnicity, and age in a sample of 2,022 3D scans.

**Methods:**

Three-dimensional (3D) body scanning technology was used to gather facial measurement data. Principal components analysis (PCA) and multivariate analysis of variance (MANOVA) were employed to determine the presence or absence of differences in measurements from 3D scans between the demographic groups.

**Results:**

Results indicated that measurements from 3D scans related to respirator fit were significantly different for all groups within each demographic category (gender, race/ethnicity, and age).

**Conclusion:**

The findings suggest that demographic factors such as gender, race/ethnicity, and age have a significant impact on facial measurements from 3D scans, which has implications for respirator fit and design considerations.

**What’s Important About This Paper?**

This study is the first to utilize a large sample of 3D facial scans to assess demographic differences in facial dimensions related to respirator fit, providing critical insights for improving respirator design and safety. The findings highlight significant variations in 3D facial measurements based on gender, race/ethnicity, and age, underscoring the need for more inclusive respirator fit models. This research offers practical implications for workplace safety by contributing to better-fitting personal protective equipment for diverse populations. Its results have the potential to influence the future design and sizing of respirators, ensuring better protection for all workers.

## Introduction

Many previous research efforts, completed by government and academic institutions, have attempted to quantify the facial anthropometrics of working, respirator-wearing populations. In 1973, researchers at the Los Alamos National Laboratory (LANL) conducted research for NIOSH which evaluated the “fit of half-mask, quarter-mask, and full-facepiece respirators” (Hack et al. 1973, p. 1). To evaluate the fit of these respirators, facial anthropometric data were collected from 200 civilian males, 40% of whom were “Spanish-American” (Hack et al. 1973, p. 5). The LANL researchers did not find differences higher than 2 mm in the means of all measured face dimensions (Hack et al. 1973). However, in 1975, Leigh (1975) found that of 1467 Dow Chemical employees (127 of whom were female), 12.6% were not represented by the LANL face panel for full respirators. This finding sparked questions regarding the gender-based generalizability of the LANL face panel. Gross and Horstman (1990) utilized 3 sizes of respirators from 3 brands (9 respirators total) to conduct quantitative fit tests of respirators on 120 civilians (60 females and 61 males) and found that 95% of the participants were able to fit using the respirators provided, though (i) no brand name was given for these respirators and (ii) the sample lacked diversity regarding age, race, and ethnicity (Gross and Horstman 1990). Oestenstad and Perkins (1992) conducted research to investigate the facial measurements of 30 females and 38 males and found that the measurements collected did not differ greatly from previous research. The diversity (regarding race, ethnicity, and age) of the sample populations in these gender-focused efforts was not found to be inclusive or representative of working populations at the time (Brazile et al. 1998).

To address limitations from previous research regarding diversity and respirator fit, subsequent research efforts sought to quantify differences in facial dimensions relevant to respirator fit between racial, ethnic, age, and gender-based groups. Brazile et al. (1998) found that face measurements related to respirator fit were significantly different between different groups in gender and race/ethnicity categories. Kim et al. (2003) conducted research to assess the association between Korean facial measurements and respirator fit factors; they found that (i) male and female Koreans had significant differences in almost all measurements and (ii) respirator fit depended on different measurements for Koreans than for Americans (Kim et al. 2003). Zhuang et al. (2010) found that face measurements related to respirator fit were significantly different “between males and females, all racial/ethnic groups, and the subjects who were at least 45 years old when compared to workers between 18 and 29 years of age” (p. 391). Luximon et al. (2010) conducted research to assess the facial anthropometric variation of Chinese women, finding that Chinese females have wider and shorter faces compared to other “cultures” (p. 1). Ball et al. (2010) found that the head shape of Chinese people differed in appearance from White people, “with a flatter back and forehead” (p. 832). Using 22 facial dimensions relevant to pilot oxygen mask design, Lee et al. (2012) found that Korean male pilots’ faces differed from both Korean male civilians’ and US male pilots’ faces, and that Korean female pilots’ faces were “significantly smaller” than Korean male pilots (p. 1927). Other important research efforts have sought to confirm that current respirators fit diverse demographic populations, such as South African people (Spies et al. 2011), Chinese people (Zhang et al. 2020), and Chilean people (Rodríguez et al. 2020).

Of particular interest in the field of anthropometrics is the rise in the popularity of 3D scanning, which offers a faster, lower-contact way to analyze facial measurements that provide more context to facial surface dimensions than manual measurements. Measurements gathered from 3D scans have been utilized in previous research regarding demographics and respirator fit (Ball et al. 2010; Lee et al. 2012). The specific aim of the present study was to assess the presence of differences in facial measurements from 3D scans related to respirator fit, based on demographic factors of gender, race/ethnicity, and age in a sample of 2,022 3D scans. This study assessed the largest sample of 3D facial anthropometrics seen in the literature to date. This study helps determine if people (within the sample population) of different gender, race/ethnicity, or age groups can be expected to have different facial measurements from 3D scans relevant to respirator fit, which has implications for respirator design, sizing, and fit for diverse workers. Three research questions guided the aim of this study:

RQ1: Are differences in measurements from 3D scans present between gender groups?

RQ2: Are differences in measurements from 3D scans present between race/ethnicity groups?

RQ3: Are differences in measurements from 3D scans present between age groups?

## Methods

3D scan data for this study were purchased from Human Solutions (Human Solutions). The company collected 3D facial scans from 2,022 participants using the handheld Artec 3D structured-light scanner (Model Eva, Senningerberg, Luxembourg). Human Solutions’ proprietary 3D scan software, Anthroscan (Version 3.6.1, Kaiserslautern, Germany), was used to collect measurements from 3D scans from each 3D scan. Self-reported gender, racial/ethnic, and age information was collected at the time of 3D scanning for each of the 2,022 participants. It is of importance to note the use of self-reported gender in lieu of a more traditional demographic variable such as biologically born sex. The University of British Columbia’s Research Equity Toolkit (Lowik et al. 2022a, b) suggests that neither sex nor gender are a perfect measurement for peoples’ identities given the psychological harm of misclassification and the transient nature of bodies and expression. In the present research, the demographic of self-reported gender was collected from subjects by Human Solutions prior to the researchers’ purchasing of the data. Sex-related data was not collected from participants, and thus, was not used in the present research analysis.

### Measurement collection

**Fig. 1.**
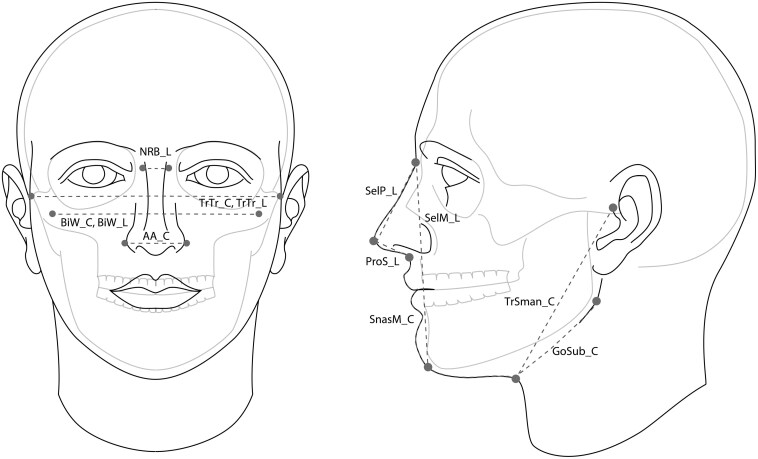
Illustration of facial measurements from 3D scans collected from each 3D scan.

Facial measurement data collection was completed by a team of 4 novice raters. The specifics of 3D facial measurement data collection for the 2,022 3D facial scans (including training, process, and missing data) are described in a previous publication (Hobbs-Murphy et al. 2024), which assessed the rater-based reliabilities of gathered measurements from 3D scans before larger 3D measurement data collection began. Figure 1 illustrates 12 facial measurements from 3D scans collected using Anthroscan software. Table 1 describes the name of each measurement, whether the measurement was collected in a linear (direct from point to point) and/or contour (over the surface of the face) fashion, and the abbreviated measurement name. These 12 measurements from 3D scans were selected from a larger sample of 27 measurements collected from the sample population in an effort to reduce the complexity of the statistical analysis (described in the Methods section below). The 12 measurements selected were chosen as most representative of respirator fit based on (i) cited relevance of measurements to respirator fit (Zhuang et al. 2007), (ii) correlation with other measurements collected (i.e. in cases of high correlation, only one measurement from the correlated set was included), (iii) rater reliability (inter- and intra-, described in Hobbs-Murphy et al., 2024), and (iv) novelty of measurement to the field of literature (i.e. does this measurement provide something new to the field of literature surrounding respirator facial anthropometrics?). Furthermore, measurement locations with a high percentage of missing values, caused by occlusions present in the 3D scan (described in Hobbs-Murphy et al. 2024), were avoided for inclusion in the selected measurements for statistical analysis in the present study. For example, head measurements such as circumference are important for respirator fit, however, the high number of missing data points (due to occlusion via head hair and hair styles) did not allow for the inclusion of head circumference variables in the present analysis.

**Table 1.** Measurement names (corresponding to Fig. 1), measurement type (linear, contour, or both), and abbreviated measurement name.

Measurement name	Measurement type	Abbreviated name
Alare to Alare	Contour	AA_C
Bizygomatic Width	Contour	BiW_C
Bizygomatic Width	Linear	BiW_L
Gonion to Submandibular	Contour	GoSub_C
Nasal Root Breadth	Linear	NRB_L
Pronasale to Subnasale	Linear	ProS_L
Sellion to Pronasale	Linear	SelP_L
Sellion to Menton	Linear	SelM_L
Subnasale to Menton	Contour	SnasM_C
Tragion to Submandibular	Contour	TrSman_C
Tragion to Tragion	Contour	TrTr_C
Tragion to Tragion	Linear	TrTr_L

### Statistical analysis

#### Principal components analysis

Principal components analysis (PCA) is a statistical dimension-reducing technique that can quantify a dataset’s variability through the calculation of principal components (PCs) (James et al. 2021; Holmes and Huber 2022). As described by anthropometric researchers Zhuang et al. (2007), “PCA defines a new coordinate system using linear combinations of the original variables to describe trends in the data.” (p. 649). Subsequent score plotting of each participant’s data based on how they relate to each principal component or the new coordinate system (Zhuang et al. 2007) can provide visual context to dataset variability (James et al. 2021; Holmes and Huber 2022). Because PCA reduces complex data to a visualizable state, it is commonly used in anthropometric research where the collection of measurement data from multiple locations for each participant typically results in a large dataset with many variables. Furthermore, PCA score plotting can help researchers visually identify important categorical trends in dataset variability. In this study, differences in variability between groups within each demographic category were analyzed using PCA score plotting. PCA and PCA score plotting were completed using R (R Core Team 2022) and packages tidyverse, readxl, extrafont, flextable, writexl, ggfortify, and scales (Horikoshi and Tang 2018; Wickham et al. 2019; Ooms 2021; Chang 2022; Gohel 2022; Wickham and Bryan 2022; Wickham and Seidel 2022).

#### Multivariate analysis of variance

Multivariate analysis of variance (MANOVA) is a statistical technique that can assess multiple continuous dependent variables (i.e. measurement variables) to determine the presence of significant differences between multiple categorical independent variables (i.e. demographic variables) (R in Action 2021). Compared to other methods of analysis of variance (ANOVA), MANOVA allows for a higher correlation between continuous variables, which is inherently present in anthropometric research (e.g. participants with wider faces tend to have wider face measurements overall). Previous research from Brazile et al. (1998) used MANOVA to assess demographic differences in respirator-relevant face measurements. In the presence of significant findings in the present study, further examination of significant differences for the 12 measurement variables was done using post hoc ANOVA testing. To complete the MANOVA and post hoc ANOVA analyses, R Studio (R Core Team 2022) and R packages tidyverse, readxl, extrafont, flextable, car, broom, and emmeans were used (Wickham et al. 2019; Chang 2022; Fox et al. 2022; Gohel 2022; Lenth 2022; Robinson et al. 2022; Wickham and Bryan 2022).

## Results

From the full sample of 2,022 subjects, missing data values for the 12 measurements from 3D scans were as follows (abbreviated measurement name from Table 1, count): SnasM_C, 236; SelM_L, 224; TrSman_C, 132; GoSub_C, 127; TrTr_C, 38; TrTr_L, 34; ProS_L, 19; AA_C, 17; BiW_C, 17; BiW_L, 17; NRB_L, 15; SelP_L, 15. Within one subject’s full data, just one or several of the above-listed data points could be missing. Because missing values cannot be used in PCA or MANOVA, participants with missing measurement values were removed from the analyses in this study. The resulting sample size was reduced from 2,022 to 1,677 participants/scan subjects. All analyses presented in this research were run using the reduced dataset of 1,677 subjects.

The racial/ethnic composition of the *n* = 1,677 subject sample is as follows: *n* = 1,040 or 62.02% White/Caucasian (referred to as White); *n* = 446 or 26.60% Black, African, or African American (referred to as Black); *n* = 84 or 5.00% Latin/Hispanic (referred to as LatinX); *n* = 81 or 4.83% Asian/Asian American (referred to as Asian). 1.55% of subjects identified as Other (*n* = 13), American Indian or Alaska Native (*n* = 6), Native Hawaiian or Pacific Islander (*n* = 3), and prefer not to say (*n* = 4). Due to small sample sizes, these 1.55% of subjects (*n* = 26) have been grouped together and will be referred to as Other in regard to their racial/ethnic category.

The gender composition of the subject sample included n=681 or 40.60% identifying as Male, and *n* = 996 or 59.40% identifying as Female/Other. The Female/Other category is comprised of *n* = 994 Female participants, *n* = 1 participant who identified as non-binary or other, and *n* = 1 participant who preferred to not share their gender. The 2 participants who fit outside the majority binary gender categorization of the sample were retained and collapsed into the Female gender category in an efforts to allow for the maximum representation of gender diversity in this research (Lowik et al. 2022).

Lastly, age was provided for each participant as an exact number. For this research, the age of the subject sample was divided into 3 categories or groups; Group limits were developed based on the youngest (18 years) and oldest (72 years) subject ages with approximately equal age spacing in each group. The Youngest group (18 to 34) is comprised of *n* = 826 subjects or 49.26%. The Mid-age group (35 to 54) comprised *n* = 777 subjects or 46.33%. The Oldest group comprised *n* = 74 subjects or 4.41%. Full demographic details of the sample population in this research in table format can be found in the Supplementary Materials for this article.

### Principal components analysis

**Fig. 2.**
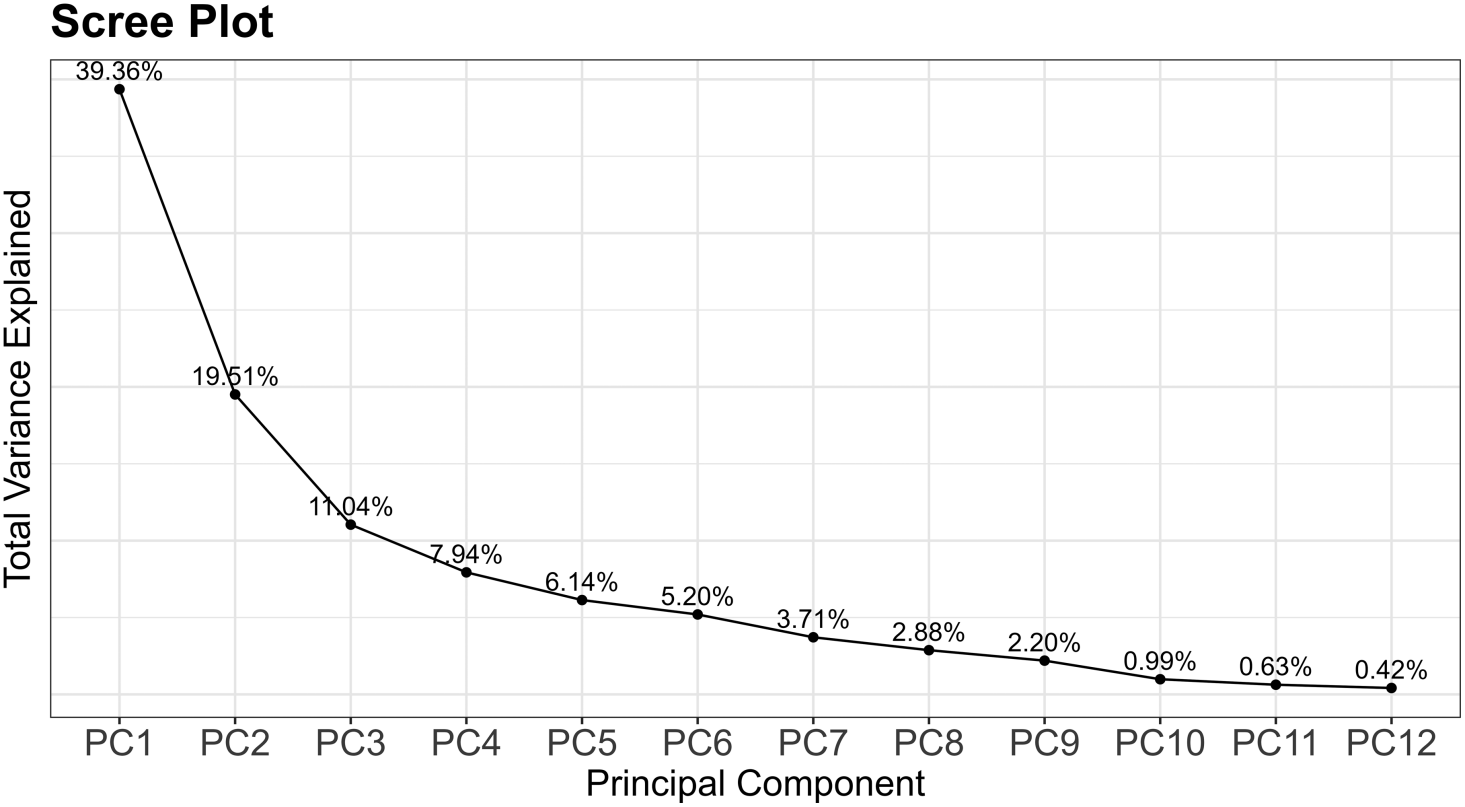
Scree plot illustrating the proportion of variance explained by each principal component.

**Fig. 3.**
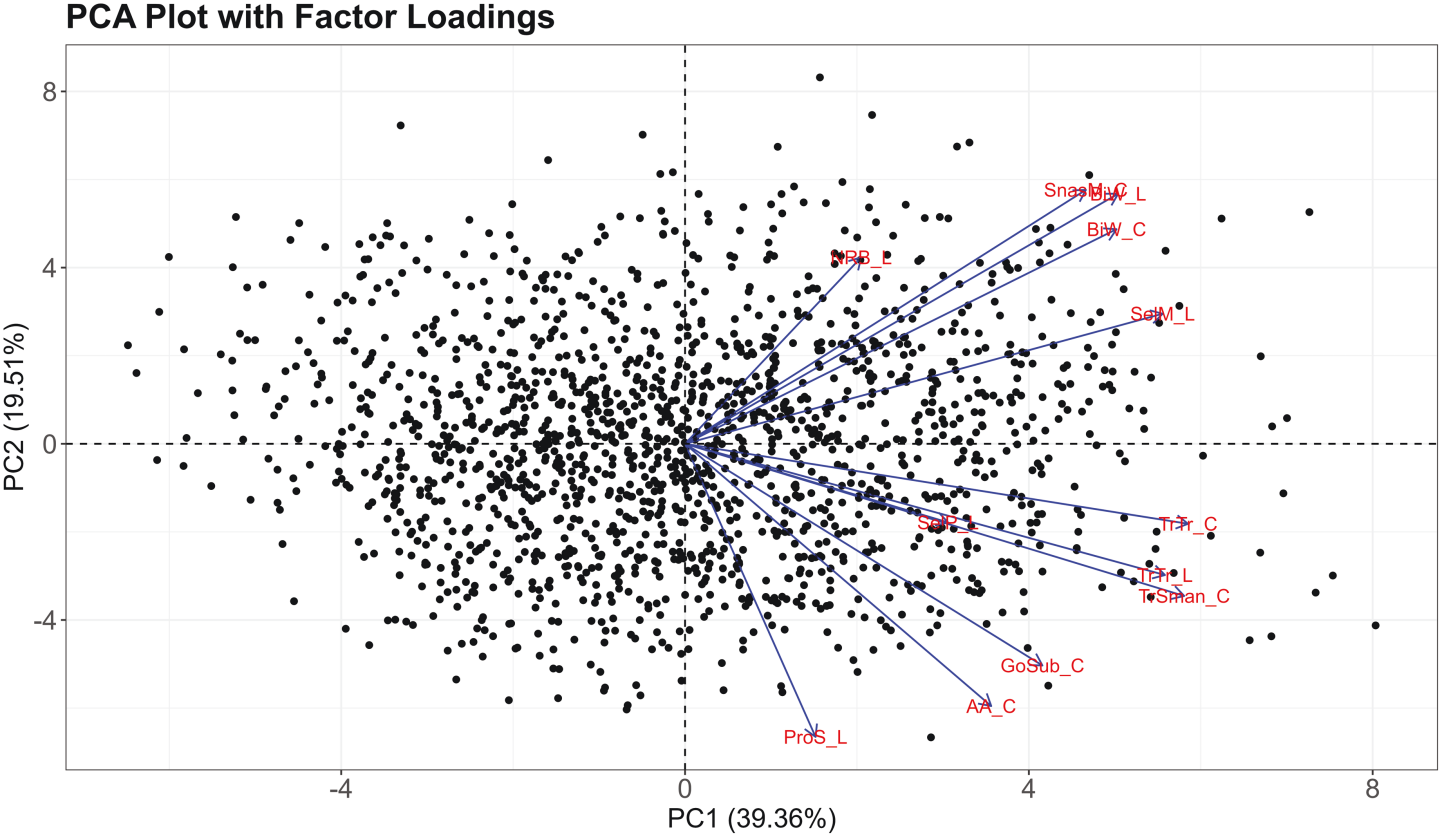
PCA score plot with factor loadings.

**Fig. 4.**
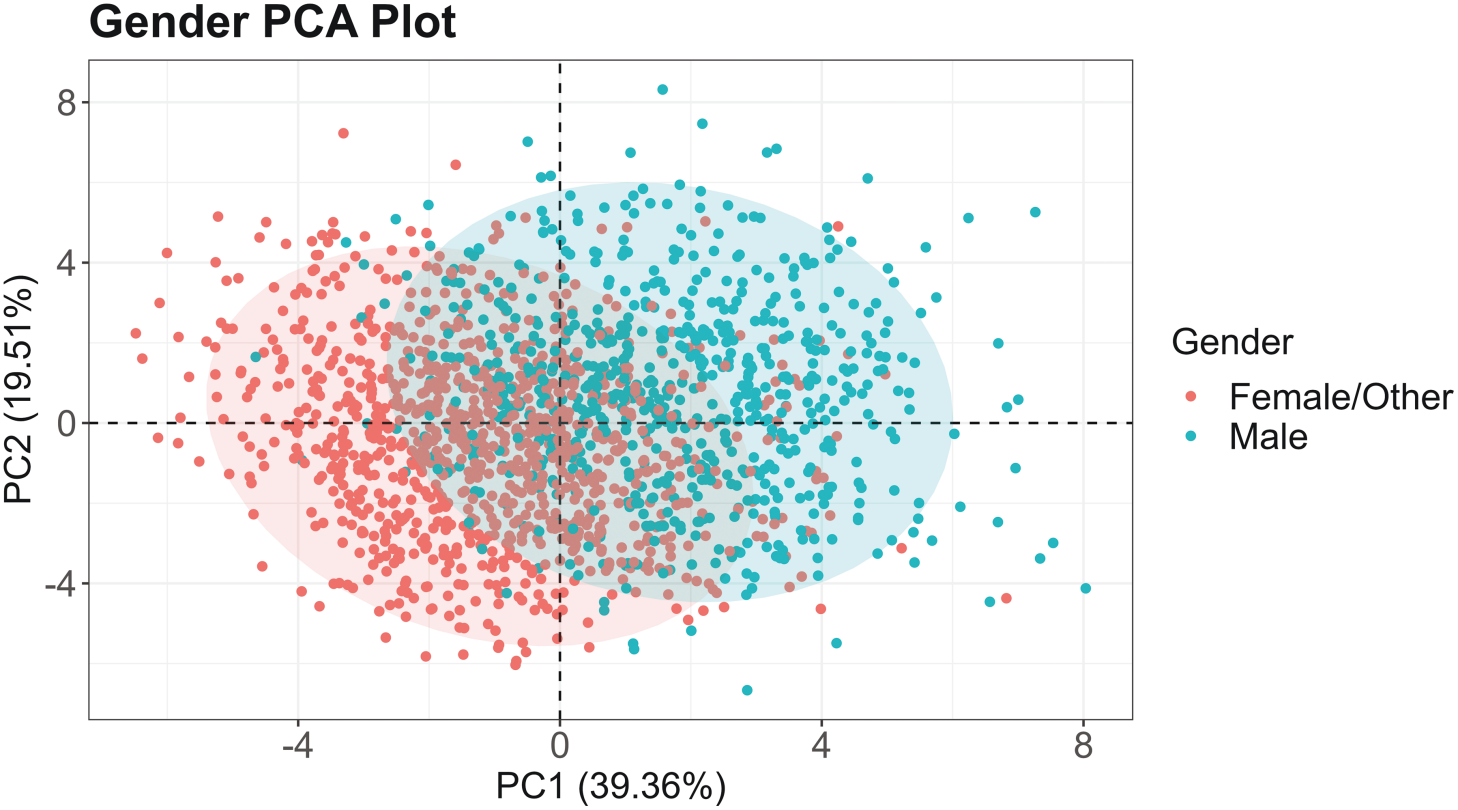
PCA score plot with gender category ellipses.

The scree plot in Fig. 2 illustrates the percentage of variability described by each principal component, with total variability described by the 12 PCs equaling 100%. Table 2 provides the factor loadings for each measurement location for PC1, PC2, and PC3, together describing 69.91% of the variability in the dataset. PC1 and PC2 described 58.87% of the variability in the dataset and were used as the new coordinate system (Zhuang et al. 2007) to plot each observation (i.e. each participant). Figure 3 illustrates a PCA score plot of the entire dataset, with factor loadings overlaid. Long line lengths (either in the positive or negative direction) indicate large factor loading, or strong variable effect on the principal components (PC1 and PC2 only). Small angles between lines on the factor loading plot indicate a positive correlation between variables. Right (90-degree) angles between lines indicate a lack of correlation. Large (180-degree) angles indicate a negative correlation; however, no negative correlations are seen in the factor loadings plot in Fig. 3. Figure 4 presents a PCA score plot with gender category ellipses, illustrating the distribution of participants along the principal components. Additional demographic PCA score plots, highlighting further demographic differences, can be found in the Supplementary Materials for this article.

**Table 2.** PCA factor loadings for Principal Component 1 (PC1), Principal Component 2 (PC2), and Principal Component 3 (PC3). Variables with the largest loadings, and therefore with the highest influence on each PC, are bolded.

Measurement name	Abbreviated measurement name	PC1	PC2	PC3
Alare to Alare Contour	AA_C	0.23	**−0.38**	−0.27
Bizygomatic Width Contour	BiW_C	0.32	0.31	−0.15
Bizygomatic Width Linear	BiW_L	0.32	**0.36**	0.06
Gonion to Submandibular Contour	GoSub_C	0.26	**−**0.32	**0.40**
Nasal Root Breadth Linear	NRB_L	0.13	0.27	0.21
Pronasale to Subnasale Linear	ProS_L	0.10	**−0.42**	**−**0.26
Sellion to Pronasale Linear	SelP_L	0.19	**−**0.11	**−0.64**
Sellion to Menton Linear	SelM_L	**0.35**	0.19	**−**0.28
Subnasale to Menton Contour	SnasM_C	0.30	**0.37**	**−**0.06
Tragion to Submandibular Contour	TrSman_C	**0.37**	**−**0.22	0.30
Tragion to Tragion Contour	TrTr_C	**0.37**	**−**0.12	0.15
Tragion to Tragion Linear	TrTr_L	**0.35**	**−**0.19	0.16

### Multivariate analysis of variance

The assumptions for MANOVA testing are independent observations, normality, homogeneity of covariances, and linear response. These assumptions were approximately satisfied within the present study’s dataset. Due to low or no representation of some demographic interactions (e.g. there were no participants with race/ethnicity as Other, gender as Male, and age as 55 to 74), a Type I additive MANOVA model was used in this work. Based on the MANOVA analysis findings, there were significant measurement differences between groups within each demographic category. In other words, people in different groups within the demographic categories of gender, race/ethnicity, and age can be expected to have one or more different facial measurements from 3D scans (of the 12 measurements assessed, Fig. 1). Post hoc ANOVA testing was done to assess which of the 12 measurements were significantly different for each demographic group. ANOVA findings (*F*-statistic, *P*-value, significance based on level *P* < 0.05) presented in Table 3 revealed that the majority of the 12 measures assessed in this research were different for people of different genders, different race/ethnicity, or different age groups.

**Table 3.** Findings from post hoc ANOVA tests (significance level = *P* < 0.05). Degrees of freedom (df) for all measurements from 3D scans: gender = 1, race_eth = 2, age_group = 4.

Measurement	Demographic factor	*F*-statistic	*P*-value	Significant?
BiW_C	Gender	210.08	<0.01	TRUE
	Race/Ethnicity	5.40	<0.01	TRUE
	Age Group	1.65	0.19	FALSE
NRB_L	Gender	8.73	<0.01	TRUE
	Race/Ethnicity	22.79	<0.01	TRUE
	Age Group	2.12	0.12	FALSE
SnasM_C	Gender	117.56	<0.01	TRUE
	Race/Ethnicity	61.24	<0.01	TRUE
	Age Group	2.65	0.07	FALSE

^*^All other facial measurements from 3D scans tested as significant across all 3 demographic factors (gender, race/ethnicity, and age group).

## Discussion

The results of the statistical analyses indicated important differences in face measurements between different groups within genders, races/ethnicities, or age categories. Table 4 compares the findings of previous studies (discussed in the Introduction) to the findings of this study. Overall, this study found differences in measurements from 3D scans related to respirator fit based on demographic factors beyond what was found in previous literature.

**Table 4.** Findings in previous literature compared to findings from this study.

Source	Findings from previous studies regarding differences in facial measurements related to respirator fit	Finding/s from this study regarding differences in facial measurements related to respirator fit
Hack et al., 1973 (LANL)	No measurement differences (<2 mm) between facial measurements of participants (*n* = 200, males only, 40% Spanish-American).	Significant measurement differences between demographic groups.
Leigh, 1975	12.6% of participants (*n* = 1467, 120 females) were not represented by LANL.	Significant measurement differences between gender groups but did not compare measurements to LANL.
Gross & Horstman, 1990	5% of participants (*n* = 120) were not able to fit 9 selected respirators.	This study did not use respirators to assess fit.
Oestenstad & Perkins, 1992	Measurements did not differ from previous research (*n* = 68).	Significant measurement differences between demographic groups but did not compare measurements to previous studies.
Brazile et al., 1998	Significant measurement differences between gender and race/ethnicity groups (*n* = 186).	Significant measurement differences between gender and race/ethnicity groups, as well as age groups.
Kim et al., 2003	Significant measurement differences between gender (male vs female) for Korean people, as well as Korean people and people of other origins (*n* = 110).	Significant measurement differences between gender and race/ethnicity groups but did not collect information about nationality.
Ball et al., 2010	Measurement differences in head shape between Chinese people and White people (*n* = 1200, males only).	Significant measurement differences in race/ethnicity groups but did not collect information about nationality.
Luximon et al., 2010	From summary statistics, measurement differences between specifically Chinese females and people of other origins (*n* = 772, females only).	Significant measurement differences between gender and race/ethnicity groups but did not collect information about nationality.
Zhuang et al., 2010	Significant measurement differences between gender (male vs. female), all racial/ethnic groups, all sampled occupations, and those aged >45 compared to those aged 18 to 29 (*n* = 3997).	Significant measurement differences between gender, race/ethnicity, and age groups but did not collect information about occupation.
Spies et al., 2011	86% of South African participants (*n* = 29) were not able to fit a size medium disposable respirator.	Significant measurement differences between race/ethnicity groups but did not collect information about nationality.
Lee et al., 2012	Measurement differences between Korean male pilots and civilians, US pilots, and Korean female pilots (*n* = 336).	Significant measurement differences between gender and race/ethnicity groups but did not collect information about nationality or occupation.
Zhang et al., 2020	Significant measurement differences between Chinese males and females (*n* = 85).	Significant measurement differences between race/ethnicity groups but did not collect information about nationality.
Rodríguez et al., 2020	Measurements of Chilean people are comparable to measurements found in previous research (*n* = 474).	Significant measurement differences between demographic groups but did not (i) collect information about nationality or (ii) compare measurements to previous studies.

The factor loadings provided in Table 2 and illustrated in Fig. 3 indicate that PC1, accounting for 39.36% of the variability in the dataset, was most influenced by measurement variables Sellion to Menton Linear, Tragion to Submandibular Contour, Tragion to Tragion Linear, and Tragion to Tragion Contour. These measurements from 3D scans are large, across-face measurements from 3D scans that indicate the overall shape and size of the face. Of note is the importance of the Tragion to Submandibular Contour measurement to PC1; this is a measurement contextualizing face length that has not been assessed by previous literature and thus offers a novel finding to this field of research.

PC2, accounting for 19.51% of the variability in the dataset, was most influenced by measurement variables Alare to Alare Contour, Bizygomatic Width Linear, Pronasale to Subnasale Linear, and Subnasale to Menton Contour. These measurements from 3D scans are generally smaller in metric length than those that influenced PC1. Compared to PC1, which captured large, positive variance, PC2 captured a more nuanced story of positive and negative variance in the dataset. The 2 largest PC2 factor loadings (provided in Table 2 and illustrated in Fig. 3) indicate that Alare to Alare Contour, a nose width measure, and Pronasale to Subnasale Linear, a nose protrusion measure, affect the overall variance in the facial measurement dataset more than the ten other measurement location variables. Notably, the factor loadings for these measurements, both of which are related to nose shape, were found to be negative. When PC2 scores (for the PCA score plots) were calculated for each observation or participant, these measurements related to nose shape are minimized by the large negative factor loadings. Furthermore, PC3, accounting for 11.04% of the variability in the dataset, was very largely negatively affected by Sellion to Pronasale, a measure of nose bridge length. The influence of nose measurements on PCA are a new finding compared to other similar research, which have tended to find PC1 related to face length and PC2 related to face width (Zhuang et al. 2007, 2010).

Based on the MANOVA testing, different groups within demographic categories of gender, race/ethnicity, and age group can be expected to have significant differences in the 12 tested facial measurements related to respirator fit. These findings show similarities and differences to previous literature findings, which are summarized in Table 4. Differences within each demographic category, including results of PCA, MANOVA, and ANOVA analyses, are discussed further below.

### Differences between gender groups

In the address of RQ1, differences in measurements from 3D scans between gender groups are discussed. Based on the gender-grouped PCA score plot (found in Supplementary Materials), gender appears to have the highest difference in variability between groups (Male versus Female/Other) out of the 3 demographic categories (race/ethnicity, gender, age group). Furthermore, the gender-grouped PCA score plot indicates that (i) male faces may be quite larger overall than Female/Other faces (PC1) and (ii) males may have slightly larger noses than those identifying as Female/Other (PC2). Based on the MANOVA findings, people of different gender groups within the analyzed sample had significant differences in at least one of the 12 analyzed facial measurements that relate to respirator fit. These results mirror those from Zhuang et al. (2010), who found gender to be the most impactful demographic factor in predicting differences in face size (compared to race/ethnicity and/or age). Post hoc ANOVA analysis (Table 3) found that all 12 measurements tested were significantly different between people of different genders. Previous relevant research efforts have not found all tested measurements to be significantly different between demographic groups of gender (Brazile et al. 1998); however, this may be attributed to smaller sample size and different measurements collected compared to the present study.

### Differences between race/ethnicity groups

In the address of RQ2, differences in measurements from 3D scans between race/ethnicity groups are discussed. The race/ethnicity-grouped PCA score plot (found in Supplementary Materials) indicated differences between race/ethnicity groups in overall face size (PC1) in order from smallest to largest (actual metric size): Asian, LatinX, White, Other, Black. However, the race/ethnicity-grouped PCA score plot illustrated minimal differences in nose size (PC2) between the 5 race/ethnicity groups. Based on the MANOVA findings, people of different race/ethnicity groups within the analyzed sample had significant differences in at least one of the 12 analyzed facial measurements that relate to respirator fit. These results mirror those from Zhuang et al. (2010), who found significant differences in face size between different race/ethnicity groups. Post hoc ANOVA analysis (Table 3) found that all 12 measurements tested were significantly different between people of different race/ethnicity groups. Previous relevant research efforts have found not all tested measurements to be significantly different between demographic groups of race/ethnicity (Brazile et al. 1998), however, this may be attributed to the smaller sample size and different measurements collected compared to the present study.

### Differences between age groups

In the address of RQ3, differences in measurements from 3D scans between age groups are discussed. The age-grouped PCA score plot (found in Supplementary Materials) indicates differences in variability between youngest (18 to 29), mid-age (37 to 54), and oldest (55 to 74) age groups. The age-grouped PCA score plot indicated some age-related differences in overall face size (PC1) and nose size (PC2): the youngest age group had the smallest faces and noses, and the oldest age group had the largest faces and noses (with the mid-age group between the two). Based on the MANOVA findings, people of different age groups within the analyzed sample had significant differences in at least one of the 12 analyzed facial measurements that relate to respirator fit. Similar to the findings of the present study, Zhuang et al. (2010) found measurement differences between face size for people in 3 age groups, although age brackets were assigned somewhat differently with the oldest group being >45. In the present study, ANOVA testing found 9 of the 12 measurement locations to be significantly different for people of different age groups. Of relevance to the literature is the finding that Bizygomatic Width Contour (a measurement indicating face width) was not significantly different for people of different age groups. Zhuang et al. (2010) found that their oldest participants (>45) had longer and narrower faces than their youngest participants (18 to 29). Therefore, results from this study contradict the results of Zhuang et al. (2010) in that older participants did not have significantly narrower faces in this study.

### Limitations

PCA cannot be used to assess statistical significance due to the lack of formal testing, thus results are open to researcher interpretation. Despite best efforts to assess PCA results in the most logical way, the interpretation of PCA results from the present study should be viewed somewhat as opinion. All anthropometric data collection and research efforts have limitations regarding the diversity of their sample population, with the present study being no exception. Due to model complexity and low representation of certain groups, interactions could not be included in the MANOVA model. By instead using an additive MANOVA model, this research could only determine the presence of significant differences between each demographic group within a single demographic category (as opposed to differences across groups, i.e. if White mid-age Females have different facial measurements than youngest LatinX males). Furthermore, previous research efforts have considered the nationality and occupation of their sample population, which this study did not. Lastly, due to the small sample size of non-binary and undisclosed gender participants (*n* = 2), they were grouped with the Female category to ensure representation; however, we acknowledge that this approach may not fully capture gender diversity within the dataset. Despite these limitations, this research contributes to the knowledge base surrounding respirator-specific facial anthropometrics from 3D scans and demographically related differences in these measurements.

### Future research

As the workforce in the United States and around the world continues to evolve, it is important that researchers continue to assess the diverse anthropometrics of relevant populations in an effort to inform the design and sizing of personal protective equipment. Future researchers may seek to include additional variables in their analyses such as subject weight, height, and crucial head measurement variables. Additionally, researchers should continue to explore anthropometric data from 3D scans in methodologically innovative ways such as assessments of 3D geometric shape variability across subject heads and faces.

## Conclusions

The present study utilized a large sample of 2,022 3D facial scans to assess demographic differences in measurements related to respirator fit. This work has practical implications for the designers who develop and size respirators, professionals who fit respirators, workers who utilize respirators in their daily work, and researchers who study facial anthropometrics (specifically in relation to respirator fit). Furthermore, this work utilized anthropometric data from 3D scans, which may have novel practical implications for designers and researchers interested in 3D scanning and anthropometrics. For example, this work found that a novel measurement related to face width (Tragion to Submandibular Contour) was able to predict a large amount of variability in the entire dataset.

In agreement with previously published research, people of different gender, race/ethnicity, or age groups had significantly different face measurements related to respirator fit. Unlike previous studies, this study found that (i) nose shape was negatively predictive of variation in the facial anthropometric dataset, (ii) all measurements tested were significantly different for different groups within gender and race/ethnicity categories, and (iii) face width was not significantly different between age groups. Future research is needed to continue to assess if diverse demographic factors have significant effects on facial measurements and facial measurements from 3D scans specifically.

## Supplementary material

Supplementary material is available at *Annals of Work Exposures and Health* online.

wxaf012_suppl_Supplementary_Material

## Data Availability

The data supporting the findings of this study are available from the first author upon reasonable request. Due to privacy and ethical restrictions, some aspects of the data may be subject to limited access.
