# Supplementary material for: Demographic differences in facial anthropometric data from 3D scans and implications for respirator fit
Source: Ann Work Expo Health. 2025 Mar 19;69(4):442–52. doi: 10.1093/annweh/wxaf012 (PMC12018073; doi:10.1093/annweh/wxaf012)
Supplement: wxaf012_suppl_Supplementary_Material [file wxaf012_suppl_supplementary_material.pdf]

DEMOGRAPHIC DIFFERENCES IN FACIAL ANTHROPMETRIC DATA FROM 3D SCANS AND IMPLICATIONS FOR RESPIRATOR FIT

Kayna Hobbs-Murphy[1], William J. Brazile[2], Kristen Morris[1], John Rosecrance[2]

[1]*Department of Design and Merchandising, Colorado State University, 1574 Campus Delivery, Fort Collins, CO, United States 80523-1574*

[2]*Department of Environmental and Radiological Health Sciences, Colorado State University, 1681 Campus Delivery, Fort Collins, CO, United States 80523-1681*

Corresponding Author: Kayna Hobbs-Murphy, [Kayna.Hobbs-Murphy@colostate.edu]

Table S1: Demographic Breakdown of Study Participants (n = 1677).

Category	Subcategory	n (%)
**Race/Ethnicity**	White	1040 (62.0%)
	Black, African, or African American	446 (26.6%)
	LatinX	84 (5.0%)
	Asian/Asian American	81 (4.8%)
	Other*	26 (1.5%)
	**Total**	**1677 (100%)**
**Gender**	Female/Other**	996 (59.4%)
	Male	681 (40.6%)
	**Total**	**1677 (100%)**
**Age Group**	18–34 (Youngest)	826 (49.3%)
	35–54 (Mid-age)	777 (46.3%)
	55–72 (Oldest)	74 (4.4%)
	**Total**	**1677 (100%)**

* “Other” includes American Indian/Alaska Native (n=6), Native Hawaiian/Other Pacific Islander (n=3), Other (n=13), and Prefer Not to Say (n=4).
** Female/Other includes Female (n=994), Non-binary/Other (n=1), and Prefer Not to Say (n=1).

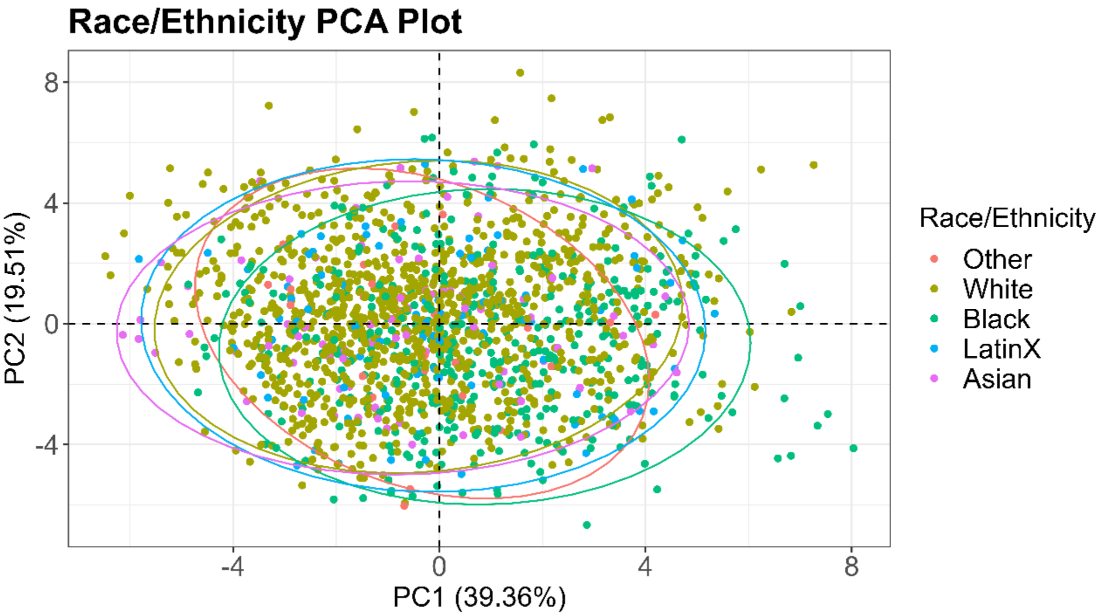

Figure S1: PCA score plot with race/ethnicity category ellipses.

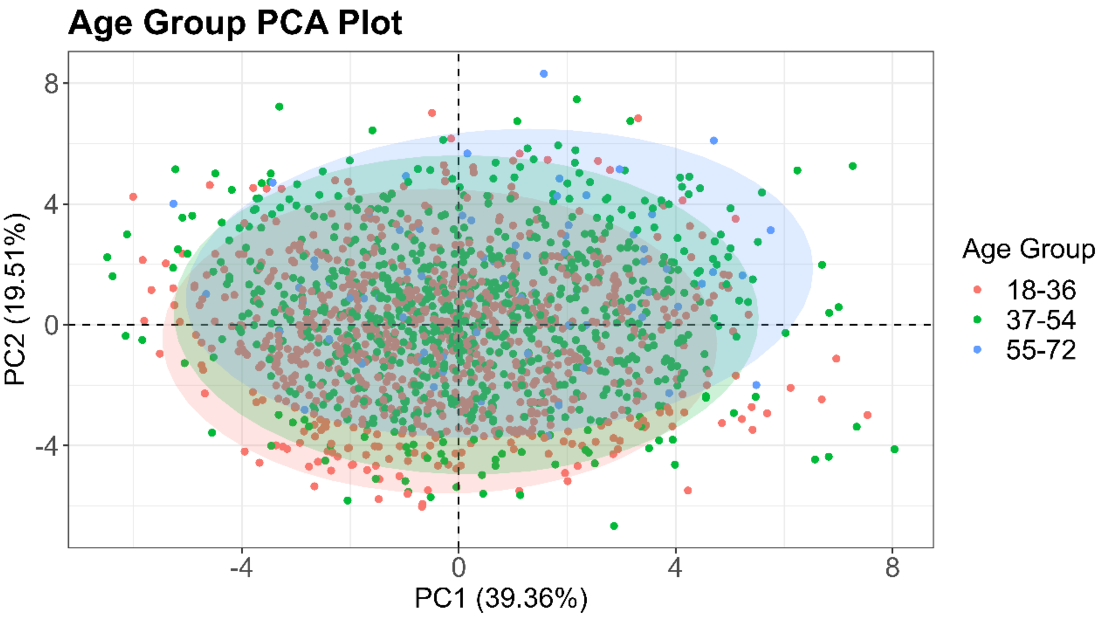

Figure S2: PCA score plot with age group category ellipses.
